# Zero-Waste Approach for Heavy Metals’ Removal from Water with an Enhanced Multi-Stage Hybrid Treatment System

**DOI:** 10.3390/ma16051816

**Published:** 2023-02-22

**Authors:** Danijela Urbancl, Darko Goricanec, Marjana Simonic

**Affiliations:** Faculty of Chemistry and Chemical Engineering, University of Maribor, Smetanova 17, 2000 Maribor, Slovenia

**Keywords:** river sediment, heavy metals, extraction process, EDTA, citric acid, natural clay

## Abstract

The aim of the work was to develop a zero-waste technological solution for hybrid removal of heavy metals from river sediments. The proposed technological process consists of sample preparation, sediment washing (a physicochemical process for sediment purification), and purification of the wastewater produced as a by-product. A suitable solvent for heavy metal washing and the effectiveness of heavy metal removal were determined by testing EDTA and citric acid. The process for removing heavy metals from the samples worked best with citric acid when the 2% sample suspension was washed over a 5-h period. The method was chosen of the adsorption of heavy metals from the exhausting washing solution on natural clay. Analyses were performed of the three main heavy metals, Cu(II), Cr(VI), and Ni(II), in the washing solution. Based on the laboratory experiments, a technological plan was prepared for the purification of 100,000 tons of material per year.

## 1. Introduction

Soils polluted with heavy metal polluted soils pose a negative impact on health and the environment due to their accumulation in the food chain. The main anthropogenic soil pollution sources are different chemicals used or produced as by-products of industrial activities, and municipal waste with harmful effect on the environment [1]. Regulation on Determination of Pollution of Agricultural Land and Forests includes quantitative estimate of what it takes to achieve standards, setting the maximum amount of pollution that a waterbody can receive without violating standards [2]. Pollutants can have adverse effects on plant growth, groundwater, crops, and permanent soil fertility [3]. Soil degradation can be divided into that caused by humans and that due to natural causes [4]. Several factors affect the metal concentration in polluted soils, such as pH, temperature, dissolved oxygen, hydrodynamic properties (water flow, amount of rain), and so on. If the concentration of oxygen falls below 7 mg/L, then the metals are released into an aquatic environment [5].

Heavy metals are a group of metals that include copper, mercury, lead, zinc, cadmium, tin, arsenic, and nickel, which are very dangerous to the environment and organisms. Numerous physicochemical and biological methods are used to remove heavy metals from contaminated soils [6,7]. Physicochemical methods include soil washing, solidification and stabilization, reduction, oxidation, dehalogenation, thermally accelerated extraction, contaminant evaporation vapor extraction of pollutants, incineration, and pyrolysis [8,9,10]. Authors have reported that a multistage process is required for optimum soil and sediment treatment. Phytoremediation predominates among the various biological methods. When chemicals are used for soil remediation, the chemicals used must not be allowed to remain in the soil, because, in some cases, they may pose the same or an even greater potential hazard to the environment as the contaminants present in the soil. Heavy metal remediation can be accomplished by removing the entire concentration of contaminants, by leaching a specific metal, or by a combination of these methods. There are various technologies for remediation of contaminated metals [11]. Depending on the site of the soil remediation, two options are distinguished, namely:

- in situ, which means that the contaminated soil is cultivated in its original place of contamination.
- ex-situ, which refers to excavating the contaminated soil from the original location to other places for subsequent restoration.

The washing of sediments and soil is ex-situ remediation, and intense stirring is required for metal removal from the soil or sediment into the aqueous phase. Several acids have been applied to aid this process, such as HCl, citric acid, and chelators like ethylene diamine tetra-acetic acid (EDTA), nitrile acetic acid (NTA), and ethylene diamine disuccinic acid (EDDS) [9,11].

The technological process of metal removal contains several stages, including preparing the sediment for washing, followed by washing and by-product wastewater treatment [12]. In the initial phase, the non-soil material is removed from the sediment, which is then passed through sieves. Smaller and finer particles contain more heavy metals than larger particles, and therefore most of our laboratory experiments were performed on smaller particles [13]. After washing, the purified soil is released into the environment and the wastewater is treated further due to its high metal content. If the treatment is not successful, further stages must be applied.

Citric acid can remove metals below the legislated limits for soil reclamation, backfilling of the lower layers of agricultural land and building land, and backfilling of mineral raw material areas for filling the soil after excavation. It was reported that the initial concentration of Zn(II) was 375 mg/kg, that of Pb(II) was 292 mg/kg, and that of Cd(II) was 340 mg/kg soil. The pH was measured at 7.15. The results of the experiments showed that the removal efficiencies were the highest by using EDTA between 50% and 60% for Zn(II), Cd(II), and Pb(II) [14].

Agricultural soil was treated using EDTA to remove metals. The main pollutants were Cd(II), Pb(II), and Zn(II). The experiments were done on the field for three weeks, and the efficiencies of removal were 80% for Cd(II), 69% for Cu(II), 73% for Pb(II), and 62% for Zn(II). On a laboratory scale, the efficiencies were 5–50% higher [15]. Overall, 48% of Pb(II) and 60% of Cu(II) were extracted from the soil if the concentration of humic solution was 2% [16]. The capacity of chitosan for Cu(II) removal was 43% at pH 3–3.5 [17].

Multistage remediation of heavy metals from sediments was done with sampling at a depth of 0.5 m [14]. Firstly, sieving was applied with 75 µm, 150 µm, 300 µm, and 600 µm meshes, and the following metal concentrations were measured in each fraction: Cd(II), Zn(II), Cu(II), As(III), and Hg(II). The concentrations of metal were the highest in the fractions below 150 and 75 µm, and in fractions above 600 µm the concentrations were much lower. In smaller particles, the metals are adsorbed strongly due to higher surface activity [18].

It was found that different clay materials mostly immobilize the metals. In the case of Zn(II) and Cu(II) the mobility was reduced, which is less toxic for the environment [19]. Clay contains exchangeable ions such as Na(I), Ca(II), and K(I), thus it is a very efficient adsorbent for the removal of heavy metals from aqueous solutions [20]. Most clay minerals are negatively charged and are used for the removal of metals from wastewater. They also have a large surface area. A literature review showed that metal adsorption could be high [21]. Waste clay has been added to road material and stabilized by using a green binder [22]. Different proportions of ceramsite were added into cement concrete and the properties were compared to raw concrete [23]. The ceramsite addition to concrete improved its mechanical strength and showed the lowest chloride ion migration coefficient.

The aim of the present work Is to study the process of heavy metal removal from polluted river sediment. EDTA and citric acid were used for the extraction of metals from the samples. Cu(II), Ni(II), and Cr(VI) were chosen, because they are harmful heavy metals which cause harmful diseases in humans and animals, and pose a threat to the environment [24]. Based on the measured concentrations of metals, the efficiency of the process was determined as a function of operating time. The novelty is demonstrated in the attempt to achieve a zero-waste metal removal system. Firstly, extraction of the metals from soil was performed, followed by natural clay treatment of the generated wastewater from the first stage. Clay with bonded metals could be used as an additive to road material, and the sediments could be released back to the environment safely.

## 2. Experimental

To determine the operating parameters for the multistage process, the experiment was divided into four main parts:

- The evaluation of the washing process procedure with EDTA for three different samples was presented in detail in our previous work [25], summarized in Section 2.2.
- Evaluation of the washing process with EDTA and citric acid for river sediment.
- Treatment of the by-product wastewater produced by washing the sediments using natural clay [26].
- Design of a multistage technological process for the removal of heavy metals from river sediments.

### 2.1. Sampling and Analyses of Samples

River sediment was sampled and analyzed from the River Drava (near Mežica, Slovenia). Mežica used to be a Zn and Pb mine, which is why monitoring of heavy metals is still being carried out. Mining stopped in 1993 due to depleted ore reserves [27]. The samples were dried at 105 °C until they had a constant weight. In the first phase, the sample was sieved into different fractions, but it was not possible to detect any difference in composition among the fractions, so the research was continued with the whole sample. The content of some typical heavy metals was determined in the raw samples. For metal determination, a solid sample was first digested with aqua regia.

Aqua regia is a mixture obtained by combining hydrochloric acid and nitric acid (V) in the ratio 3:1. The acid mixture is very corrosive and unstable, and therefore it must be used immediately after preparation. 1.5 g of the sample was weighed. The samples were then transferred to a 100 mL flask and moistened slightly with millipore water and dissolved in a mixture of 21 mL of 30% HCl and 7 mL of 65% HNO_3_. The solution was heated and boiled at 110 °C for the next two hours. The cooled mixtures were then filtered into a 100 mL flask. The filter residue was washed with millipore water. The samples in the flasks were made up to the 100 mL mark with millipore water and mixed well at 250 rpm. The contents of Cu(II), Cr(VI), and Ni(II) in the samples were determined using ICP-MS following the modified SIST EN ISO 17294-2 (2016) [28]. The efficiency of the sediment purification was verified by analyzing the Cu(II) content, since its content was the highest in the raw sediment.

### 2.2. Evaluation of the Washing Procedure with EDTA and Citric Acid for River Sediment

The washing procedure with EDTA and citric acid for river sediment was divided into three parts. The optimal washing time of the sediment samples and the optimal solvent were determined in the first phase. The second phase was to analyze the process of adsorption of metal ions on clay for purification of by-product wastewater after sediment washing. The third phase was devoted to the development of a hybrid model for the removal of heavy metals from river sediments.

#### 2.2.1. Washing Procedure

The sediment washing procedure was performed on a laboratory scale. 5 g of sediment was weighed into six 100 mL flasks. A citric acid solution was added to three flasks, prepared by first pouring 25 mL of 0.1 M citric acid into a flask, and then diluted with millipore water up to the 100 mL mark. An EDTA solution was added to the other three flasks, also prepared in a 100 mL graduated cylinder, into which 18 mL of 0.1 mol/L EDTA was first poured, and then diluted to 100 mL with millipore water. All six samples were mixed, with a sediment mass concentration of 20 g/L. To determine the effect of leaching time, the sediment samples were mixed for different times. Two sediment samples (one with EDTA, the other with citric acid) were mixed for 4 h, another two for 5 h, and the last two for 6 h. The pH of the sediment suspension samples was measured before and after stirring. The sample suspensions were filtered after stirring. The settling time of the particles was shorter for the citric acid samples.

The concentration of Cu(II) in the liquid phase was determined by the Inductively coupled plasma mass spectrometry method (ICP-MS). The equations used to calculate the effect of Cu(II) removal from the sediment are given below.

Equation (1) shows the calculation of the Cu(II) concentration in the filtrate (c, mg/L):

c = (c_o_ × V_sol_)/V_f_ (1)

where c_o_ is the initial concentration (mg/L), V_f_ represents the filtrate volume (mL), and V_sol_ is the volume of the solution (mL) [8].

Equation (2) shows the calculation of the Cu(II) concentration (c_r_) removed from the sediment (mg/kg d.m.):

c_r_ = (c_o_ × V_sus_)/m_s_ (2)

where m_s_ is the mass of the sediment (g) and V_sus_ is the washing suspension volume (L)

Equation (3) shows the calculation of the Cu(II) removal efficiency (RE) from the sediment:

RE (%) = (c_o_ − c_r_)/c_o_ × 100% (3)

#### 2.2.2. Treatment of By-Product Wastewater after the Washing Process

After the sediment has been cleaned of heavy metals, the by-product effluent is discharged back into the river. In Slovenia, the by-product effluent must comply with the limit values of the parameters according to the Decree on the Emission [29].

The measured value for Cu(II) does not meet the statutory limits for discharge into a river. Among the most favorable processes for removing pollutants is adsorption. Therefore, we analyzed a wastewater treatment process using the method of adsorption of metal ions on natural clay to determine the amount of clay required to meet the limits for metal discharge into a river.

#### 2.2.3. Clay Characterization Methods

The clay originated from the surroundings of Celje, Slovenia. The clay was analyzed with emphasis on those elements which have the most effect on adsorption efficiency. The zeta potential was determined in order to assess the charge. The zeta potential measurements were based on the mobility of particles in an electric field. The value of potential above 30 V means a stable colloid, and below unstable. Agglomerates are formed due to Van der Waals forces. A Malvern Zetasizer nano sizer series was applied for the zeta potential and particle size determination. The measurements were made at room temperature. The average particle size of the natural clay was determined based on dynamic light scattering. The porosity and chemical composition were determined with an electron microscope (Quanta 200 3D). Before the analysis, an Au layer was applied onto the surface to achieve suitable electrical conductivity and stability of the sample. The X-Ray Diffraction technique (XRD) was applied for determination of the chemical composition.

The removal efficiency was calculated with Equation (3) (see above) and the adsorption capacity q_m_ using Equation (4):

q_m_ (mg/g) = (c_os_ − c_ts_)/m (4)

where m is the mass of the clay, and co and c_t_ in the solution (Equation (3)) are replaced by c_os_ and c_ts_ in the sediment.

### 2.3. Designing the Hybrid Technological Process for the Removal of Heavy Metals from River Sediments

A technological process for the removal of heavy metals from river sediments was developed based on the experimental results. First, the following values were chosen:

- Amount of raw sediment: 43,000 t d.m./year.
- Required volume of washing solution based on the laboratory experiments: 5 g sediment in 100 mL.
- Sediment composition: The average heavy metal content of the sediment as given in Table 1.

Citric acid was used to wash the sediment. The required dose of the acid with a concentration of 5 mol/L was 0.25 m^3^/t d.m., and the reaction time was 5 h.

## 3. Results

### 3.1. The Analysis Results of Raw Samples

The concentration of each heavy metal in the samples was determined using the analytical method ICP-MS. The results of the analyses are shown in Table 1. The last line of the table summarizes the maximum values of the parameters in the excavated soil for soil remediation, in backfilling the lower layers of agricultural land and building land, and in backfilling areas with mineral raw materials to fill the soil after excavation according to [30].

The highest concentration (400 mg/kg d.m.) was measured for Cu(II). In addition, Cr(VI) was also detected in the sample. Based on the obtained results. further analyses were performed, and the purification effects were evaluated by analyzing the Cu(II) content, since it was highest in the raw sediment.

#### Clay Characterization

The average zeta potential in a sample of clay at 25 °C was −22.2 mV. The negative value showed the high probability of the attraction of positive ions. Since the studied cations had a positive charge, high adsorption efficiency was expected. Additionally, the literature review showed that cation adsorption could be high [22].

**Figure 1:**
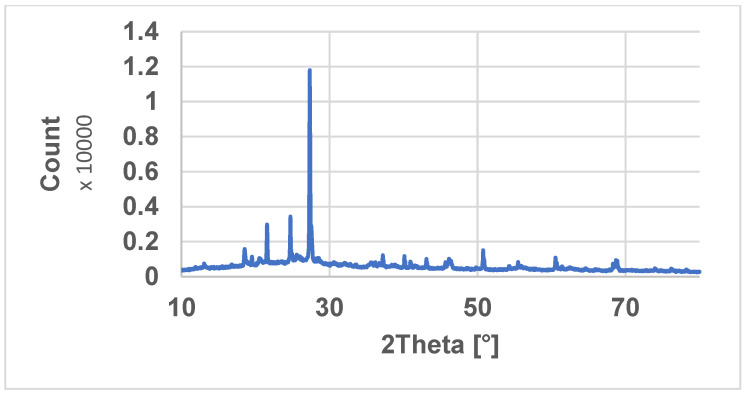
XRD pattern of natural clay.

**Figure 2:**
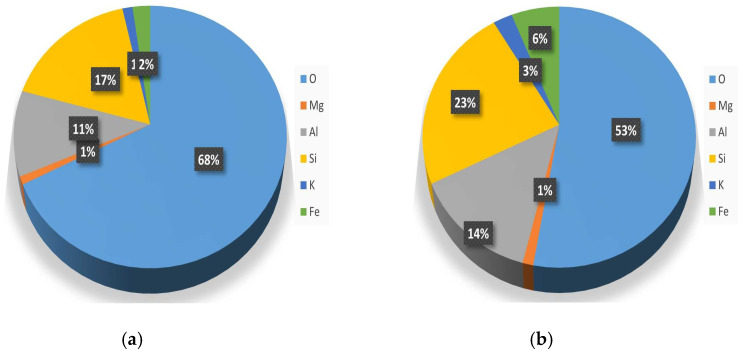
The atomic (**a**) and mass (**b**) share of the elements in natural clay.

Particle size distribution analysis after three measurements showed the average value of 1285 nm for the clay samples. Figure 1 represents the XRD pattern of natural clay, and Figure 2 shows the mass and atomic share of the elements.

The mineralogical composition of ceramsite was identified through X-ray diffraction (XRD) measurements. As shown in Figure 1, the XRD patterns of clay exhibit sharp peaks, indicating the existence of crystalline phases. The most intense peaks in the XRD patterns are located at around 27°, and belong to the ceramsite, in good agreement with the literature [24]. Similar results were also obtained in another study [31].

**Figure 3:**
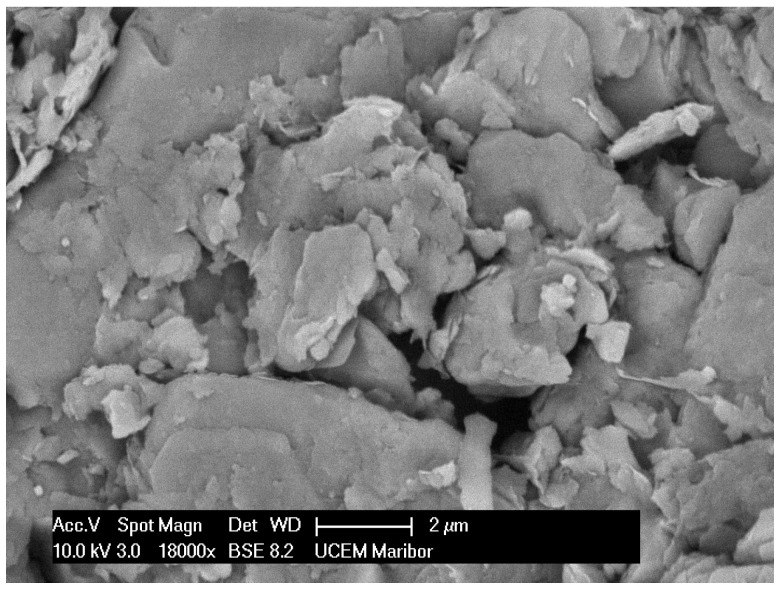
18,000-time magnification of clay.

The clay is a very porous material with some holes between the layers, which is seen from the SEM image in Figure 3.

### 3.2. Results of the Washing Procedure with EDTA and Citric Acid for River Sediment

Sediment containing increased amounts of heavy metals, such as Cu(II), Ni(II), and Cr(VI), must be treated properly. The methods should be chosen to achieve the quality as specified in the literature [30]. The maximum allowed values in sediment are Cu(II)-60 mg/kg d. m., Ni(II)-20 mg/kg d. m., and Cr(VI)-0.5 mg/kg d. m. The conditions must be achieved for the use of construction material prepared from treated or untreated, source, or waste mineral raw materials.

Prior to washing, the sediment was sieved into three fractions, particles of size > 500 µm, particles of size between 500 µm and 160 µm, and particles of size < 160 µm, to determine the fraction composition. Published scientific research articles indicate that the content of heavy metals in sediment is increased for particles smaller than 600 µm. Our sample sediment contained the most particles smaller than 160 µm. Table 2 shows the fractional composition of the sample sediment as determined by sieve analysis.

Heavy metal content analyses were performed in the individual fractions, but no differences in concentrations between the individual fractions were observed, so sieving analysis was not required. The washing (extraction) experiment of 5 g of sediment in 100 mL EDTA at original pH (denoted pH_before_) and in citric acid at an acidic pH solution was performed at different reaction times, namely, 4, 5, and 6 h. The results of the pH measurements before and after washing (denoted pH_after_) are shown in Table 3. The pH in the EDTA solution was alkaline, determined between 8.6 and 8.8. In the citric acid it increased from an initial pH of 2.7 to above 3.5. Alkaline compounds are likely to be released from such sediments. Table 4 shows the Cu(II) removal efficiencies using EDTA and citric acid as a function of washing time (t).

From Table 4 it is seen that, after 4 h, the removal of Cu(II) with EDTA is comparable with that at 5 h contact time. After 6 h the removal decreased using EDTA, and this might be attributed to the less strong EDTA-Cu bond compared with other metals, such as EDTA-Ni. Low efficiencies are the consequence of less attraction of EDTA-Cu compared with other metals, such as EDTA-Ca [28]. With citric acid, the efficiency stabilized after 4 h, and this offers the greatest potential for its use as a chelating agent [14]. Table 5 shows the values of heavy metals before and after treatment of the sediment with citric acid.

The metal content in sediment was washed successfully with citric acid. The Cu(II), Ni(II), and Cr(VI) concentrations all fell below the maximum regulated limits. More specifically, the metal concentration values agreed with the legislated ones for excavated soil for soil remediation, in backfilling the lower layers of agricultural land and building land, and in backfilling areas with mineral raw materials to fill the soil after excavation. As such, the washed sediment could be used for all these agricultural and construction processes.

### 3.3. Results of By-Product Wastewater Treatment

After the process of extracting heavy metals and removing sediments, by-product wastewater was generated, the quality of which did not meet the levels for direct discharge into the environment. The generated water must thus be treated further, and so the process of the adsorption of metal ions on natural clay was studied.

The results of metal adsorption onto clay after 5 h are presented in Table 6. It shows the removal efficiency (η), and the amount of clay needed for metal removal at certain initial metal concentrations. The mass of required clay could be calculated with the data from Table 6. For removal of Cu(II), Ni(II), and Cr(VI), the required mass of clay was determined at 1.0 g clay per L of washing wastewater by initial concentrations in the second row in Table 6.

The experimental data for all isotherms in single and binary ion systems complied with the Langmuir and Freundlich models (not shown) [21]. The clay showed higher affinity to Cu(II) than Cr(VI) for both single and binary ion systems under similar experimental conditions. According to the literature, Cr(VI) 88%, Cu(II) 82%, Ni(II) 86% removals can be expected from wastewater [32]. Similar results were obtained for Cr(VI), and even better for Cu(II) in the present study [33].

Considering the effects of treatment, the results show that the content of heavy metals in the treated wastewater is below the maximum legislated value, which means that the treated wastewater is suitable for discharge into the environment.

### 3.4. Designing the Hybrid Technological Process for the Removal of Heavy Metals from River Sediments

**Figure 4:**
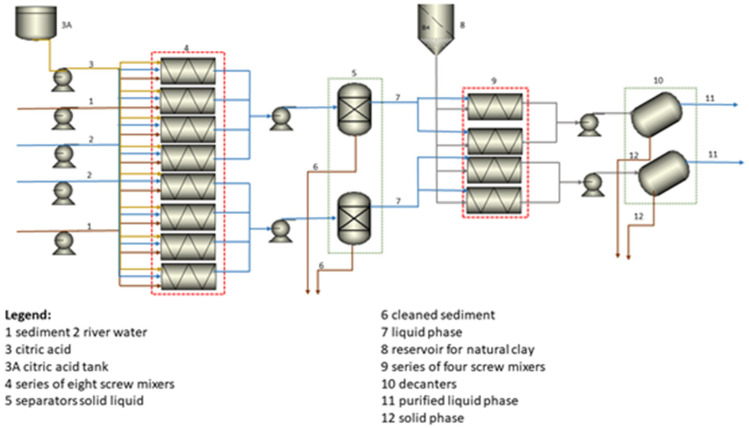
Process scheme.

The process scheme is seen from Figure 4. A solution for the hybrid removal of heavy metals from river sediments was developed based on the results of laboratory experiments on washing with citric acid. In addition to the laboratory experiments (Table 6), the following effects are summarized:

- 99% removal of Cu(II)
- 80% removal of Cr(VI)
- 75% removal of Ni(II)

For the treatment of the by-product wastewater, we considered natural clay with a particle size of about 1 µm in an amount of 1 g/L of wastewater. The working process of sediment treatment was planned in two lines. The treatment of 43,000 tons d.m. waste sediment is carried out 345 days a year, 24 h a day. According to the laboratory results, the following removal of heavy metals is expected:

- 99% removal of Cu(II)
- 80% removal of Cr(VI), which are both close to the reported values [34], while Ni(II) remained below the limit value.

Two lines with three subassemblies were predicted in the technological scheme. The sediment cleaning process was divided into the following technological subassemblies:

- sediment preparation, removal of metals from the sediment
- sediment washing, and
- by-product wastewater treatment.

#### 3.4.1. Sediment Preparation

The excavated wet river sediment was transported by crane to six mixing reactors where the process of washing metal ions with the citric acid solution took place. The dehydrated whey was transported by a conveyor belt to the storage facility. The treated effluent was discharged into the environment after the whole process. Clay was used as a substitute for aggregates in the production of road material.

#### 3.4.2. Sediment Washing

The sediment was brought into the reactors by crane. An appropriate amount of river water and citric acid was pumped into the reactors by the pump, so that the resulting suspension had a mass concentration of 20 g/L. The thus-prepared solution was mixed with the help of air. The sediment washing process consists of two processes: extraction and sedimentation. The extraction of metal ions into the solvent consists of three phases: filling (takes 1 h), mixing (takes 4 h), and draining (takes 1 h). The total reaction time is 5 h, as the reaction takes place half an hour before the end of filling and continues for half an hour in the emptying phase. The suspension is then pumped into a settling tank. During the settling process, the process of neutralization with NaOH also takes place. The neutralization process increases the pH of the suspension if required. The filtrate flows by gravity into the reactors for the adsorption process. The settled sediment is pumped by a screw pump to a belt press, where it is concentrated and then transported to the purified sediment storage facility. Prior to disposal in the wild, the treated sediment must be analyzed, to determine if it is suitable for disposal or if it must be returned to the washing process.

#### 3.4.3. By-Product Wastewater Treatment

The clear part, which is contaminated with heavy metals, flows by gravity into the reactors for the adsorption process. In the reactors, an appropriate amount of clay is added to the wastewater so that the mass concentration of the clay suspension is 1.0 g/L. The suspension is aerated for better adsorption. The process of by-product wastewater treatment consists of three stages: filling (takes 1 h), mixing (takes 4 h), and draining (takes 1 h). The total reaction time is 5 h, as the reaction takes place half an hour before the end of filling and continues for another half an hour in the draining phase. After the purification process, the suspension is pumped into a mechanical thickening device (consisting of a dewatering table and a belt press). The dewatered clay is transported to the storage facility via a conveyor belt. After the treatment process, the treated effluent is returned to the river. Clay can be used as a road material. The leaching experiments were performed by adding 100 mL of deionized water to 1 g of whey. The concentrations of metals were measured at the beginning and the end of the experiment. The results showed that the concentration of all three metals in a water solution remained the same after 72 h of stirring. Waste clay could be used as an additive in concrete [24], or together with a green binder for road material [23].

## 4. Conclusions

The sustainable environment treatment processes were studied for heavy metal removal from sediments. It was found that the particle size had no effect on the metal concentration. Washing with citric acid showed better performance compared to EDTA, while 60% of Cu(II) was removed with EDTA and 80% was removed with citric acid after 4 h. After separation of the cleaned sediment, it was released into the environment, while the wastewater produced as a by-product required further treatment. Clay, composed mainly of silicon and aluminum oxides, proved to be a satisfactory choice, as up to 99% removal of Cu(II) and lower removal of Cr(VI) were achieved at 80%, while the concentration of Ni(II) remained below the limit of 1 mg/kg d.m. due to the adsorption process. Thus, the quality was high enough to discharge the wastewater into the environment. The results show the potential for a zero-waste hybrid system.

## Figures and Tables

**Table 1:** Concentration of heavy metals in the raw samples (mg/kg d.m.).

Sample	cCu(II)	cCr(VI)	cNi (II)
sediment	400	2	40
limited values	40	30	30

**Table 2:** Sediment composition.

	Particle Size	Mass Fraction (%)
fraction	>500 µm	26
fraction	160–500 µm	<1
fraction	<160 µm	74

**Table 3:** Data for pH measurements in solutions.

t (h)		EDTA Solution pH	Citric Acid pH
4	pH_before_	8.8	2.7
	pH_after_	8.6	3.5
5	pH_before_	8.8	2.7
	pH_after_	8.6	3.7
6	pH_before_	8.8	2.7
	pH_after_	8.7	3.8

**Table 4:** Results of Cu(II) removal from sediment.

	t (h)	c_o_ (mg Cu(II)/kg d.m.)	c_rem_ (mg Cu(II)/kg d.m.)	η (%)
EDTA	4	400	160	60
	5	400	160	60
	6	400	140	65
Citric acid	4	400	88	78
	5	400	80	80
	6	400	80	80

**Table 5:** Metal content in the sediment before and after treatment in (mg/kg d.m.).

	c_Cu_	c_Cr_	c_Ni_
Raw sediment	400	2	40
Treated sediment	80	0.5	<1

**Table 6:** Adsorption results.

Metal	Sediment Solution (mg/L)	Treated Sample Solution (mg/L)	Removal Efficiency (%)	Adsorption Capacity (mg Metal/g Clay)
Cu(II)	80	1	99	79
Ni(II)	<1	<1	-	-
Cr(VI)	1.5	0.3	80	1.2

## Data Availability

Data is unavailable due to privacy.

## Nomenclatures

c	(mg/L)	concentration of metal in the filtrate
c_os_	(mg/kg d. m.)	initial amount of metal in the raw sediment
c_ts_	(mg/kg d. m.)	amount of metal in the raw sediment after time t
c_o_	(mg/L)	initial metal content in the wastewater
c_s_	(kg/m^3^)	mass concentration of sediment in the suspension
c_t_	(mg/L)	metal concentration in the wastewater after adsorption treatment
c_r_	(mg/kgd.m.)	metal concentration removed from the sediment
m	(g)	mass of the clay
ms	(g)	sediment mass
pH_before_	-	pH in the solutions before washing
pH_before_	-	pH in the solutions after washing
q_m_	(mg/g)	adsorption capacity
RE	(%)	efficiency
t	(h)	time
V_f_	(mL)	filtrate volume
V_sol_	(mL)	solution volume
V_sus_	(mL)	washing suspension volume
